# Childhood trauma and subclinical hypomania in early adulthood: A genetically informative study

**DOI:** 10.1192/j.eurpsy.2025.10117

**Published:** 2025-10-10

**Authors:** Irene Gonzalez-Calvo, Angelica Ronald, Laura Havers, Erin Lawrence, Mark Taylor, Georgina Hosang

**Affiliations:** 1 Centre for Psychiatry and Mental Health, Wolfson Institute of Population Health, Faculty of Medicine & Dentistry, Queen Mary University of London, London, UK; 2 Department of Psychology, Faculty of Health and Medical Sciences, University of Surrey, Surrey, UK; 3 Department of Medical Epidemiology and Biostatistics, Karolinska Institutet, Stockholm, Sweden

**Keywords:** bipolar disorder, childhood trauma, gene–environment interplay, genetic risk, hypomania, twin study

## Abstract

**Background:**

There is preliminary evidence that childhood trauma (e.g., abuse) is associated with subclinical hypomania reported in adolescence. These findings need replicating in early adulthood, as clinical conditions emerge, and the mechanisms underlying this association need elucidating. This study aimed to examine the magnitude of shared genetic and environmental underpinnings of the association between childhood trauma with hypomanic symptoms and high-risk status for bipolar disorder (BD) using a twin design. Gene–environment correlations and interactions between childhood trauma and polygenic scores (PGS) for psychiatric and neurodevelopmental conditions were also investigated.

**Methods:**

Childhood trauma was reported using the Avon “Life at 22+” questionnaire by 8,464 individuals from a community twin sample. Self-reported hypomanic symptoms were assessed using the Mood Disorder Questionnaire at age 26 by 7,748 participants. PGS for psychiatric and neurodevelopment conditions were derived from independent published discovery samples.

**Results:**

Childhood trauma was significantly associated with hypomanic symptoms (β = 0.23, 95% CI: 0.20–0.25) and being at high-risk for BD (OR = 1.77, 95% CI: 1.59–1.98). These associations were strongly influenced by genetic factors (bivariate heritability range: 0.51–0.90). Gene–environment correlations were found between childhood trauma and the PGS for six conditions: Major Depressive Disorder (MDD), schizophrenia, Attention-Deficit Hyperactivity Disorder, anxiety disorders, Post-Traumatic Stress Disorder, and BD II (β range = −0.19–0.11). The MDD-PGS was found to significantly interact with childhood trauma in hypomania (β = 0.01, *p < .05*).

**Conclusions:**

The associations between childhood trauma and subclinical hypomania and high-risk for BD were partially attributed to shared genetic factors. These associations were also moderated by MDD-PGS. Gene–environment correlations were detected between childhood trauma and polygenic vulnerability to psychiatric and neurodevelopmental conditions. The etiology of hypomania and BD is likely the result of a confluence of genetic and environmental factors, and research in this area should account for potential genetic confounding.

## Introduction

Hypomania (symptoms include irritability and elevated mood) is a defining feature of bipolar disorder (BD) [1], but subclinical hypomania (i.e., symptoms that do not meet the diagnostic criteria) is common in the general population. Though often transient and benign, subclinical hypomania can have serious consequences, ranging from life dissatisfaction and suicidality to the later development of psychopathology, including BD [2–4]. For instance, up to 10% of adolescents have been identified as being at high-risk for BD (based on the number, clustering, and impact of hypomanic symptoms) [2]. The study of subclinical phenotypes (e.g., hypomania) in the general population can be useful for understanding the developmental pathways of associated disorders (e.g., BD), which is critical for advancing prevention and treatment efforts [5]. Little is known about subclinical hypomania’s etiology, although this is a growing area of investigation [6].

Childhood trauma (e.g., emotional or physical maltreatment) is an important risk factor to consider, given its strong link with BD and other mental illnesses [7,8]. There is emerging evidence that childhood trauma is associated with adolescent subclinical hypomania [9]. These findings need to be replicated, especially considering hypomania at ages beyond adolescence, when psychopathology becomes more prevalent at clinical levels [10]. Because BD typically manifests between the ages of 15– 24 years [11], early adulthood constitutes a key stage to focus on the study of hypomania. Beyond associations, it is important to illuminate the etiological influences underlying the childhood trauma–hypomania link to clarify causality and guide better-targeted intervention.

### Heritability and gene–environment interplay

While childhood trauma can be considered an environmental risk factor, it is partly genetically influenced, with up to 62% of its variance attributed to genetic factors [12–14]. Furthermore, childhood trauma is linked to genetic predisposition to neurodevelopmental and psychiatric conditions, as measured by polygenic scores (PGS: risk estimates based on common genetic variants), including autism, attention-deficit/hyperactivity disorder (ADHD), post-traumatic stress disorder (PTSD), major depressive disorder (MDD), and schizophrenia [14–17]. Other studies have found a significant *negative* association with the BD PGS, with individuals with this genetic vulnerability reporting less childhood trauma [18]. These patterns provide evidence of possible gene–environment correlations (rGE), whereby one’s genetic predisposition can influence exposure to specific environments [19].

There is also evidence of gene–environment interactions (environmental and genetic effects being dependent on each other; GxE) [20] with childhood trauma in hypomania-related phenotypes. Specific polymorphisms (e.g., Val^66^Met in the brain-derived neurotrophic factor gene) have been reported to increase vulnerability to childhood trauma’s effect on subclinical psychosis [21], and the PGS for ADHD and BD have been found to moderate the impact of childhood trauma on BD [22,23]. Negative GxE effects have also been found; individuals reporting high trauma levels and with a low BD PGS presented a more unstable form of BD in a clinical sample study [18]. No gene–environment interplay studies have focused on subclinical hypomania, but similarly complex patterns are expected given its genetic overlap with BD [24], its genetic association with the conditions correlated with childhood trauma (ADHD, schizophrenia and MDD) [3], and, more broadly, based on evidence that psychiatric symptoms and life impairment are underpinned by a common cross-trait genetic factor [25].

### Study aims

The overall objective of this study was to investigate the association between childhood trauma and subclinical hypomania in early adulthood using a genetically informative approach, addressing three aims. Firstly, the phenotypic association between childhood trauma and subclinical hypomania was examined. Secondly, the degree of genetic and environmental overlap between childhood trauma and subclinical hypomania was estimated using bivariate twin models. Thirdly, the interplay between childhood trauma and PGS for nine relevant psychiatric and neurodevelopmental conditions (e.g., ADHD and BD) was investigated, focusing on rGE and GxE effects on subclinical hypomania. To fully capture this phenotype, we conceptualize subclinical hypomania in two ways: i) as a continuous trait (symptom count) and ii) as a categorical construct (individuals at high-risk for BD based on established classifications).

## Methods

### Participants

Participants were members of the Twins Early Development Study (TEDS) [26–28], a longitudinal prospective community sample of twins born in England and Wales (1994–1996) identified through the Office of National Statistics. We analyzed data from two data collection waves: ages 21 (N = 8,464; 63% female) and 26 years (N = 7,748; 65% female). Only participants with data available at both timepoints (N = 6,473) were included in analyses. Participants were excluded if they had specific medical conditions and/or extreme perinatal outliers, in line with standard TEDS procedures [27]. TEDS has ethical approval from King’s College London Ethics Committee (Reference: PNM/09/10–104). Informed consent was obtained from all participants at each wave.

### Materials

#### Childhood trauma

***Childhood trauma*** was assessed retrospectively when participants were 21 years old using eight self-report items derived from the Avon Longitudinal Study of Parents and Children “Life at 22+” questionnaire (Supplementary Table 1) [29]. Using this measure, participants reported how frequently they experienced different types of childhood emotional/physical abuse (e.g., “how often did an adult push, grab, or shove you?”), on a scale of “Never” (0) to “Very often” (4). Total scores range from 0 to 32.

#### Subclinical hypomania

***Subclinical hypomania*** was measured at age 26 using the self-reported Mood Disorder Questionnaire (MDQ) [30]. The MDQ consists of 13 yes/no items examining hypomanic symptoms [1]. The MDQ is one of the best-validated instruments for youth BD[31] and has shown good sensitivity (0.73) and specificity (0.90) for identifying bipolar spectrum disorders [32]. We categorized individuals as high-risk for BD using the criteria employed in the Genetic Links to Anxiety and Depression (GLAD) study [33], which is slightly less conservative than the MDQ criteria [32], based on the following:

1. *Number of symptoms*
  - 7 of the 13 symptoms were reported, or
  - 1 elation symptom and at least 3 of any of the other symptoms, or
  - 1 symptom of irritability and any other 4 symptoms.
2. *Symptoms clustered in the same period*, and
3. *Reported moderate/severe impairment* as a consequence.

#### Genome-wide *polygenic* scores

DNA samples were obtained from saliva and buccal cheek swabs. Genotyping was carried out by TEDS researchers on Affymetrix GeneChip 6.0. or HumanOmniExpressExome8v1.2. [34]. More genotyping information is available elsewhere [35]. PGS were derived from genome-wide association studies (GWAS) and were calculated using LDpred or LDpred-2. PGS summarize trait-associated effect sizes of individual genetic variants (weighted sum of condition-associated alleles). PGS for nine relevant psychiatric and neurodevelopmental conditions were used in this study: BD (overall, BD I and BD II) [36], MDD [37], schizophrenia [38], PTSD [39], autism [40], anxiety [41], and ADHD [42]. PGS were selected due to their previously reported association with childhood trauma, subclinical hypomania, BD, and/or other genetically overlapping conditions [15, 18, 36, 43, 44].

#### Additional *measures*

Subclinical hypomania at age 16 was measured using the self-report Hypomania Checklist-16 (HCL-16; Supplementary Materials) [45]. Hypomania data at both time points were available for 1,901 participants. Participants assessed at age 16 were part of the Longitudinal Experiences and Perceptions Project within TEDS. LEAP has ethical approval from King’s College London Ethics Committee (Reference: HR/DP-20/21–22060).

### Data analysis

Analyses were pre-registered on the Open Science Framework (https://osf.io/pvqda/) prior to receiving access to the dataset [46]. All analyses were performed using R (Version 2022.12.0 + 353 (2022.12.0 + 353)). Phenotypic variables were transformed using square root transformation techniques to meet the assumption of having a normal distribution for twin modelling. Sex was included as a covariate in all models. The Benjamini–Hochberg procedure (false discovery rate) was used to correct for multiple testing (at *p* ≤ .05) within each research question.

### Phenotypic analyses

The effects of childhood trauma on hypomanic symptoms and high-risk for BD were tested using linear and logistic regressions, respectively, using standardized scores. Analyses were implemented as doubly robust generalized estimating equations (GEE; drgee package) [47] to account for related individuals in the sample and calculate robust standard errors. *Sensitivity analyses* were used to further examine these associations while controlling for the influence of hypomania at age 16 years, since there is evidence that adolescents who display hypomania-related symptoms (e.g., impulsivity and irritability) are at increased risk of experiencing both childhood trauma [48] and later hypomania [2]. Hypomania at age 16 (N = 2,943, 57% female) was assessed as part of the Longitudinal Experiences And Perceptions project (LEAP) [49].

### Twin analyses

The classic twin design allows to decompose phenotypic variance and covariance into genetic and environmental influences using data from monozygotic (MZ) and dizygotic (DZ) twins. This method is based on the principle that MZ twins share 100% of their genetic influences (compared with DZ twins, who share approximately 50%), both MZ and DZ twins share all their common environment (i.e., factors in the same family), and are exposed to non-shared environmental factors unique to the individual, contributing toward differences between both MZ and DZ twin pairs. A detailed description of this method can be found elsewhere [50]. *Univariate structural equation twin models* were used to estimate additive genetic (A), non-additive genetic (D), shared environmental (C), and non-shared environmental (E) contributions to childhood trauma, hypomanic symptoms, and high-risk for BD. Liability threshold models were used for high-risk for BD, given the categorical nature of this variable. *Bivariate twin models* were then fitted to investigate the causes of covariation between childhood trauma and i) hypomanic symptoms (continuous model) and ii) high-risk for BD (joint categorical–continuous model). Specifically, an ACE model was used to estimate the proportion of covariance between childhood trauma and hypomanic symptoms explained by genetic (bivariate heritability) and environmental factors (bivariate shared environment and bivariate non-shared environment), as well as the genetic (r_a_) and environmental correlations (r_c_ and r_e_) between these phenotypes. These correlations reflect the extent to which the genetic and environmental influences on childhood trauma overlap with those on subclinical hypomania. Based on evidence of non-additive genetic effects, a bivariate ADE model was fitted for childhood trauma and high-risk for BD. Twin analyses were performed in OpenMx (v2.21.11), using the method of maximum likelihood estimation. In line with standard behavioral genetics procedure, the effects of sex and age were regressed out, and analyses were conducted using standardized residuals.

### Polygenic score analyses

PGS analyses were performed using GEE (accounting for sample relatedness, as described above). All models were adjusted for the first ten principal components (PCs) of ancestry, genotyping chip and batch, and sex. First, **rGE** were estimated using univariable linear regressions testing each PGS’s association with childhood trauma using the total sample. A multivariable linear regression model was subsequently fitted, including all 9 PGS as predictors of childhood trauma. In line with previous studies [51, 52], all analyses were repeated separately for the high-risk for BD and control groups to determine whether rGE were specific to either one.

Second, **GxE** were examined using both multiplicative and additive models, fitted separately for each PGS. **Multiplicative GxE models** test whether the combined effect of each PGS and childhood trauma differs from the product of their individual effects (i.e., relative risk). Multiplicative interactions were tested using linear (hypomanic symptoms) and logistic regressions (high-risk for BD/control status) [51–53]. The main effects of the PGS and childhood trauma and the interaction between the PGS and childhood trauma were included as the predictors, controlling for sex, the first 10 PCs, the interaction term between each PC and the PGS, and the interaction term between each PC and childhood trauma [51,54]. **Additive GxE models** test absolute risk. Additive interactions were tested using linear regressions on both the number of hypomanic symptoms and high-risk for BD/control status, with the PGS x childhood trauma interaction as the predictor, controlling for the same covariates used in the multiplicative models [51–53].

## Results

A description of the sample is presented in Table 1. Males reported significantly more hypomanic symptoms (M = 3.40, SD = 3.48) than females (M = 2.94, SD = 3.39; β = 0.07, SE = 0.09, *p* < .0001). Sex was not significantly associated with being at high-risk for BD (OR = 1.00, 95% Confidence Intervals [CI]: 0.80–1.27, *p* = .95). The average total score of childhood trauma did not differ significantly by sex (β = 0.02, SE = 0.02, *p* = .21). Childhood trauma descriptive statistics can be found in Supplementary Table 1.

**Table 1.** Sample description

Characteristic	Data collection waves		
	Age 16 years* [hypomania assessment] (N = 2943)	Age 21 years [childhood trauma assessment] (N = 8464)	Age 26 years [hypomania assessment] (N = 7600)
Age in years (M, SD)	17.1 (0.88)	22.85 (0.88)	26.4 (0.90)
Female	1700 (57%)	5364 (63%)	5076 (65%)
White ethnicity	2722 (93%)	7916 (93%)	7269 (94%)
Zygosity	1046 (35%) MZ	3158 (37%) MZ	2859 (37%) MZ
	911 (31%) SS DZ	2722 (32%) SS DZ	2520 (32%) SS DZ
	996 (34%) OS DZ	2584 (31%) OS DZ	2369 (31%) OS DZ
Retrospective childhood maltreatment score (M, SD)		5.30 (4.55)	
Hypomanic symptoms (M, SD)	7.16 (2.62)	-	3.10 (3.42)
Hypomania score above cut-off	1389 (48%)	-	1344 (17%)
Negative impact or reaction of symptoms^1^//Moderate or severe impairment^2^	1065 (36%)^1^	-	564 (7%)^2^
Symptom duration of 2 days or more^1^//2 or more symptoms^2^	598 (20%)^1^	-	1573 (20%)^2^
High-risk for BD	241 (8%)^a^	-	348 (4.5%) ^b^

Abbreviations: M (Mean), SD (standard deviation), N (number), MZ (monozygotic), SS DZ (same-sex dizygotic), OS DZ (opposite sex dizygotic), BD (bipolar disorder).

*Note:* See ^a^age 16 and ^b^age 26 hypomania measures for an explanation of how the high-risk groups were calculated, including information on the cut-off scores. *Data included in sensitivity analyses.

^1^ Characteristics measured at age 16 years. ^2^ Characteristics measured at age 26 years.

### Phenotypic associations between childhood trauma and subclinical hypomania

Childhood trauma (total score and each item) was significantly associated with hypomanic symptoms and increased odds of being at high-risk for BD at age 26 (Table 2). All associations remained significant in sensitivity analyses, which adjusted for hypomania at age 16.

**Table 2.** Association between childhood trauma, hypomanic symptoms, and high-risk for bipolar disorder

Childhood trauma	Hypomanic symptoms at age 26					
	Adjusted for sex			Adjusted for hypomanic symptoms at 16		
	β (95% CI)	B (95% CI)	*P*	β (95% CI)	B (95% CI)	*P*
Total score	0.23 (0.20–0.25)	0.28 (0.25–0.31)	**<.00001**	0.25 (0.20–0.29)	0.31 (0.25–0.37)	**<.00001**
Shout at you	0.18 (0.15–0.20)	0.56 (0.48–0.64)	**<.00001**	0.22 (0.17–0.26)	0.68 (0.53–0.83)	**<.00001**
Say hurtful things or insult you	0.19 (0.16–0.21)	0.34 (0.29–0.39)	**<.00001**	0.21 (0.16–0.26)	0.38 (0.29–0.47)	**<.00001**
Push/grab/shove you	0.16 (0.13–0.18)	0.32 (0.27–0.37)	**<.00001**	0.17 (0.12–0.22)	0.34 (0.24–0.44)	**<.00001**
Smack you	0.13 (0.10–0.16)	0.25 (0.20–0.30)	**<.00001**	0.16 (0.11–0.21)	0.30 (0.20–0.40)	**<.00001**
Punish you in a cruel way	0.17 (0.15–0.20)	0.33 (0.28–0.38)	**<.00001**	0.18 (0.13–0.23)	0.35 (0.25–0.44)	**<.00001**
Threaten to hit you	0.14 (0.12–0.17)	0.35 (0.29–0.41)	**<.00001**	0.15 (0.10–0.19)	0.36 (0.24–0.48)	**<.00001**
Actually hit you	0.15 (0.12–0.17)	0.43 (0.35–0.50)	**<.00001**	0.15 (0.10–0.19)	0.43 (0.29–0.57)	**<.00001**
Hit you so hard that it left you with bruises or marks	0.15 (0.12–0.17)	0.47 (0.39–0.55)	**<.00001**	0.13 (0.09–0.18)	0.44 (0.29–0.58)	**<.00001**

	High-risk for BD at age 26					
	Adjusted for sex			Adjusted for high-risk for BD at 16		
	OR	95% CI	*P*	OR	95% CI	*P*
Total score	1.77	1.59–1.98	**<.00001**	1.73	1.43–2.10	**<.00001**
Shout at you	1.84	1.58–2.15	**<.000001**	1.92	1.46–2.52	**<.00001**
Say hurtful things or insult you	1.67	1.44–1.94	**<.00001**	1.62	1.23–2.13	**.0007**
Push/grab/shove you	1.62	1.43–1.84	**<.00001**	1.51	1.19–2.92	**.0007**
Smack you	1.43	1.23–1.67	**<.00001**	1.42	1.06–1.90	**.02**
Punish you in a cruel way	1.61	1.42–1.83	**<.00001**	1.59	1.25–2.03	**.0002**
Threaten to hit you	1.43	1.29–1.58	**<.00001**	1.40	1.18–1.68	**.0002**
Actually hit you	1.46	1.34–1.60	**<.00001**	1.39	1.18–1.63	**<.00001**
Hit you so hard that it left you with bruises or marks	1.42	1.30–1.54	**<.00001**	1.39	1.19–1.63	**<.00001**

Abbreviations: β (Standardized estimates); CI (Confidence Intervals); B (unstandardized estimates); OR (Odds Ratio).

*Note*: *P*-values are reported FDR-adjusted (Benjamini–Hochberg procedure).

The significance of bold values are indicated the values of *p* ≤ .05.

### Genetic and environmental influences on childhood trauma and subclinical hypomania

Twin model assumptions were met (i.e., mean and variance differences were not statistically significantly influenced by twin order or zygosity). Univariate twin correlations for childhood trauma, hypomanic symptoms, and high-risk for BD are presented in Supplementary Table 2. The genetic and environmental estimates from the univariate twin models are shown in Supplementary Table 3. These models did not provide a significantly worse fit when compared with their corresponding saturated models.

Bivariate cross-twin cross-trait (CTCT) correlations for childhood trauma and hypomanic symptoms (Supplementary Table 2) showed that MZ CTCT correlations were larger than DZ CTCT, suggesting that their covariance was influenced by A factors. MZ CTCT correlations were slightly less than the phenotypic association (0.25, 95% CI: 0.22–0.28), indicating E influence. C influences were indicated by the DZ CTCT correlations being greater than half the MZ CTCT correlations. For childhood trauma’s association with high-risk for BD, CTCT correlations suggested A, E, and D effects, because DZ correlations were less than half the MZ correlations.

#### Childhood trauma and hypomanic symptoms

**Figure 1.**
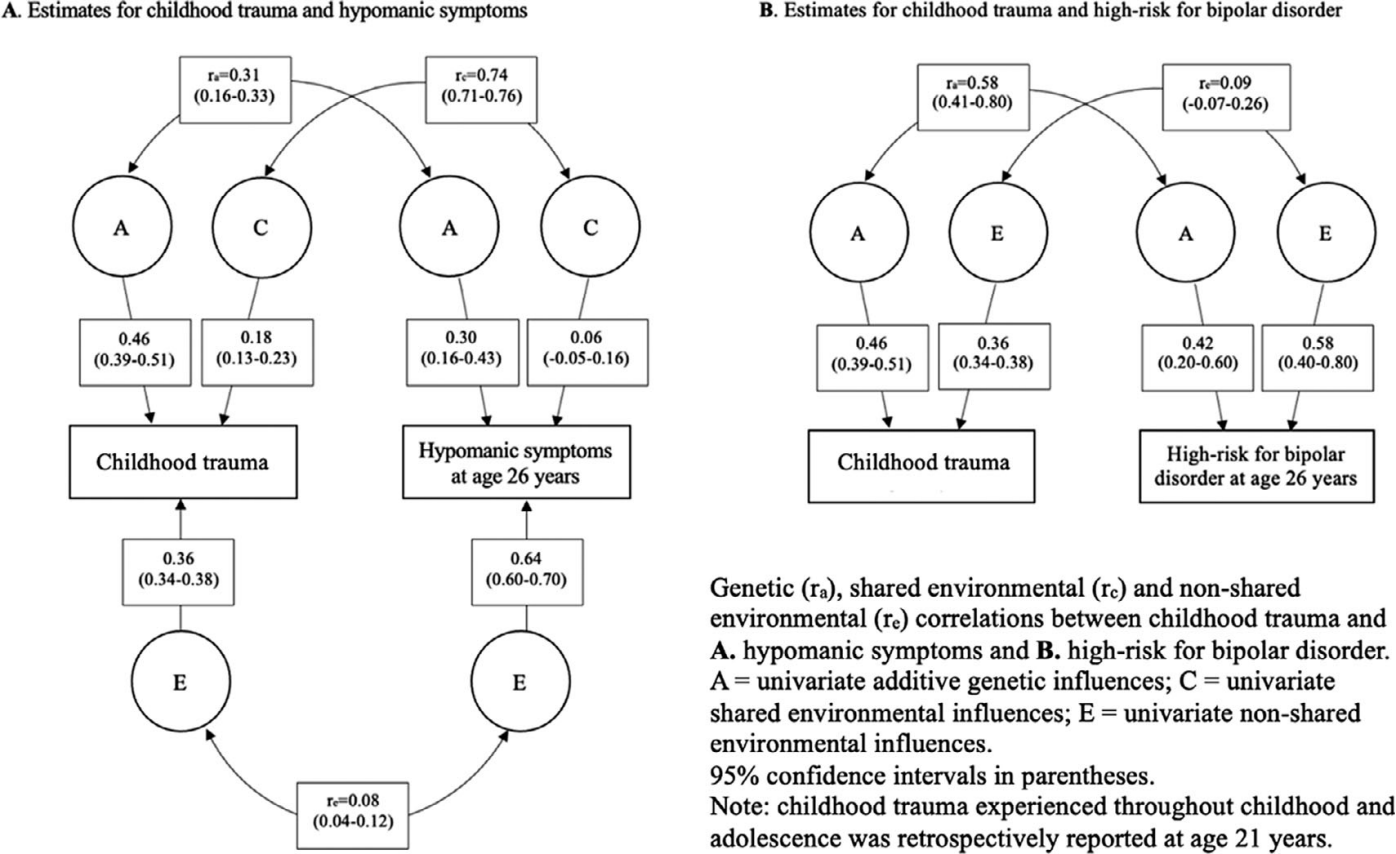
Genetic and environmental univariate estimates and bivariate correlations for childhood trauma and subclinical hypomania (number of symptoms and high-risk for bipolar disorder).

In line with the CTCT correlations for childhood trauma and hypomanic symptoms, results from the bivariate model showed that the ACE model fitted the data better than the nested models (Supplementary Table 4). The association between childhood trauma and hypomanic symptoms was mostly explained by genetic (51%) and shared environmental influences (32%). However, univariate shared environmental estimates for hypomanic symptoms showed that CIs overlapped with zero and thus lacked statistical significance. The correlation estimates showed a moderate degree of genetic overlap (r_a_ = 0.31), a large shared environmental overlap (r_c_ = 0.74), and a small non-shared environmental overlap (r_e_ = 0.08) between these phenotypes (Figure 1).

#### Childhood trauma and high-risk for bipolar disorder

While the CTCT correlations indicated A, D, and E effects, the bivariate AE model was the best fitting. However, all models provided a significantly worse fit than the saturated model (Supplementary Table 4). The association between childhood trauma and high-risk for BD was largely explained by genetic effects (90%), with a moderate genetic overlap (r_a_ = 0.58). A small non-shared environmental correlation was detected (r_e_ = 0.09), but CIs overlapped with zero (Figure 1).

### Interplay between childhood trauma and polygenic scores

#### Gene–environment correlations between polygenic scores and childhood trauma

In the overall sample, childhood trauma was significantly positively correlated with five of the nine PGS examined in univariable models: MDD, schizophrenia, ADHD, anxiety, and PTSD. A similar pattern was detected when multi-PGS models were utilized, except for the anxiety PGS. When analyses were repeated for the group at high-risk for BD, there was evidence of a negative rGE between childhood trauma and the BD-II PGS, and a positive rGE with the MDD PGS, but only in the multi-PGS model (Table 3).

**Table 3.** Gene–environment correlations between polygenic scores and childhood trauma and gene–environment interaction effects between childhood trauma and polygenic scores on hypomanic symptoms and high-risk for bipolar disorder

	Childhood trauma total score								
	Total sample(N = 4538)			High-risk for BD at age 26(N = 194)			Control(N = 4344)		
Gene–environment correlations	β	95% CI	*P*	β	95% CI	*P*	β	95% CI	*P*
**Polygenic score**									
***Univariable models***									
Bipolar disorder	0.03	0.003–0.06	.05	−0.09	−0.27–0.11	.60	0.03	−0.0004–0.07	*.06*
Bipolar disorder I	0.03	−0.003–0.05	.09	−0.02	−0.23–0.17	.83	0.03	−0.001–0.07	*.07*
Bipolar disorder II	0.03	−0.002–0.06	.09	−0.14	−0.33–0.05	.57	0.03	−0.009–0.06	*.14*
**MDD PGS**	**0.07**	**0.04–0.10**	**<.00001**	0.29	0.08–0.51	.07	**0.06**	**0.03–0.09**	***.004***
**Schizophrenia**	**0.05**	**0.03–0.08**	**.0004**	0.04	−0.14–0.23	*.82*	**0.06**	**0.03–0.09**	***.01***
**ADHD**	**0.11**	**0.08–0.13**	**<.00001**	0.08	−0.08–0.25	*.59*	**0.10**	**0.07–0.13**	***<.00001***
Autism	0.02	−0.005–0.05	.10	0.02	−0.18–0.22	*.85*	0.03	−0.00003–0.07	*.06*
**Anxiety**	**0.06**	**0.03–0.09**	**.0004**	0.12	−0.10–0.34	*.59*	**0.05**	**0.01–0.08**	***.01***
**PTSD**	**0.07**	**0.04–0.10**	**.00001**	0.12	−0.06–0.29	*.57*	**0.05**	**0.01–0.08**	***.01***
***Multi-PGS model***									
Bipolar disorder	−0.005	−0.04–0.03	.75	−0.21	−0.41–−0.001	*.05*	−0.002	−0.04– 0.04	*.15*
Bipolar disorder I	−0.002	−0.093–0.06	.90	−0.04	−0.26–0.18	*.73*	0.005	−0.03–0.04	*.79*
**Bipolar disorder II**	−0.0003	−0.02–0.06	.99	**−0.19**	**−0.37**–**0.007**	***.04***	0.003	−0.04–0.04	*.99*
**MDD**	**0.03**	**0.003–0.06**	**.03**	**0.33**	**0.10–0.56**	***.004***	0.02	−0.02–0.06	*.92*
**Schizophrenia**	**0.03**	**0.002–0.06**	**.04**	0.06	−0.15–0.27	*.58*	0.04	0.01–0.08	*.27*
**ADHD**	**0.09**	**0.06–0.12**	**<.00001**	−0.03	−0.20–0.15	*.76*	**0.08**	0.05–0.12	***.02***
Autism	0.02	−0.01–0.05	.19	−0.00003	−0.20–0.20	*.99*	**0.03**	**−0.003**–**0.07**	***<.00001***
Anxiety	0.03	−0.002–0.06	.06	0.07	−0.15–0.28	*.54*	0.02	−0.01–0.06	*.17*
**PTSD**	**0.05**	**0.02–0.08**	**.002**	0.06	−0.12–0.25	*.50*	0.03	−0.005–0.06	*.10*

	Childhood trauma x polygenic score					
	Hypomanic symptoms at age 26			High-risk for BD at age 26		
Gene–environment interactions	β	95% CI	*P*	OR	95% CI	*P*
**Multiplicative Interactions**						
Bipolar disorder	−0.002	−0.01–0.006	.82	0.91	0.80–1.03	.34
Bipolar disorder I	−0.001	−0.01–0.007	.85	0.95	0.84–1.08	.70
Bipolar disorder II	−0.004	−0.01–0.002	.43	0.87	0.77–0.98	.18
**MDD**	**0.01**	**0.006–0.01**	**.01**	1.16	1.01–1.34	.18
Schizophrenia	0.0005	−0.008–0.009	.91	0.97	0.83–1.13	.75
ADHD	0.003	−0.005–0.01	.64	0.95	0.84–1.07	.70
Autism	−0.004	−0.01–0.003	.45	0.98	0.87 .1.10	.75
Anxiety	0.005	−0.002–0.01	.43	1.02	0.89–1.17	.75
PTSD	0.008	−0.004–0.02	.27	1.09	0.97–1.23	.34

Additive Interactions	β	95% CI	*P*	β	95% CI	*P*
Bipolar disorder	−0.03	−0.07–0.0002	.22	−0.003	−0.01–0.01	.70
Bipolar disorder I	−0.04	−0.07–−0.002	.22	–0.002	−0.01–0.006	.70
Bipolar disorder II	−0.02	−0.05–0.02	.47	−0.005	−0.01–0.003	.12
**MDD**	0.01	−0.02–0.05	.33	**0.01**	**0.005–0.02**	**.02**
Schizophrenia	−0.03	−0.06–0.006	.47	−0.001	−0.01–0.01	.98
ADHD	−0.02	−0.05–0.02	.47	0.002	−0.006–0.01	.70
Autism	0.02	−0.01–0.05	.47	−0.003	−0.01–0.004	.70
Anxiety	0.01	−0.02–0.04	.47	0.006	−0.002–0.01	.34
PTSD	0.02	−0.01–0.05	.47	0.009	0.0003–0.02	.12

*Note:* P-values are reported FDR-adjusted (Benjamini–Hochberg procedure); β = Standardized beta. OR = Odds ratio Two multi-PGS models were fitted (one including the overall BD PGS and the other the BD I PGS and BD II PGS) to avoid multicollinearity.

The significance of bold values are indicated the values of *p* ≤ .05.

#### Gene–environment interactions between childhood trauma and polygenic scores on subclinical hypomania

The MDD PGS was found to significantly interact with childhood trauma on a multiplicative level in its influence on hypomanic symptoms, and additively on its effect on high-risk for BD (Table 3). No other significant GxE were detected.

## Discussion

To our knowledge, this is the first study to test the association between childhood trauma and subclinical hypomania using a genetically sensitive design in a non-clinical sample. Childhood trauma was significantly associated with hypomanic symptoms and being at high-risk for BD at age 26. We found evidence of substantial genetic influence. Additive genetic effects explained 51 and 90% of childhood trauma’s covariance with hypomanic symptoms and high-risk for BD, respectively. Moreover, moderate to strong genetic correlations between childhood trauma with hypomanic symptoms (0.31) and high-risk for BD (0.58) were observed. Next, the interplay between childhood trauma and PGS for psychiatric and neurodevelopmental conditions was examined to elucidate the specific genetic factors and the nature of their relationship with childhood trauma. Childhood trauma was significantly positively correlated with five PGS (i.e., MDD, schizophrenia, ADHD, anxiety, and PTSD), and negatively with the BD II PGS. GxE effects were detected between childhood trauma and the MDD PGS on both hypomanic symptoms (multiplicative) and high-risk for BD (additive).

### Childhood trauma and subclinical hypomania

Our findings showing the effect of self-reported childhood trauma on subclinical hypomania in early adulthood extend those of a recent study reporting a link between parent-rated childhood maltreatment and adolescent (hypo)mania [9]. Our sensitivity analyses indicated that, despite some influence, these associations were not entirely due to earlier hypomania. The strongest effects were detected for emotional abuse relative to physical maltreatment, mirroring the patterns found for hypomania-related phenotypes, including other subclinical symptoms (e.g., psychotic experiences) and BD [55–57]. Possible mechanisms may include emotional trauma’s impact on brain development, particularly emotion regulation (e.g., cortical thinning) [58], which is a key feature of mood disorders [59]. Research in the area of neurocognition may be advantageous for clarifying the nature of these associations.

### Genetic and environmental overlap between childhood trauma and subclinical hypomania

A novel finding from the present study is the moderate genetic overlap between childhood trauma and hypomania. These results are in line with those for related forms of psychopathology (e.g., broadly defined emotional disorder and depression) [60, 61]. The influence of shared genetic factors was particularly prominent in the context of high-risk for BD. This suggests that the same genes responsible for individual differences in both measures of subclinical hypomania (particularly more severe cases) may also underlie the development of characteristics that increase the risk of experiencing (i.e., active rGE) and/or evoking (i.e., evocative rGE) traumatic childhood events, such as adverse parental reactions. For instance, children and adolescents who present impulsive or irritable behaviors are more likely to experience conflict with caregivers [48, 62]. These behaviors are more prevalent – and severe – among those at high-risk for BD [2].

Another innovative finding from this study is that bivariate twin modelling suggested common shared environmental effects on childhood trauma’s association with hypomanic symptoms. This finding may reflect true shared environmental mediation, although it could also be indicative of passive rGE; children of parents with mental health problems (e.g., BD) have greater risk of inheriting a genetic predisposition to psychopathology (e.g., mood disorder traits), but parents affected by mental illness are also more vulnerable to adversity, which additionally increases the risk of offspring childhood trauma [63, 64]. However, the shared environment had a very small influence on hypomanic symptoms, and CIs overlapped with zero. Further research is necessary to expand our results; adoption studies using registry data could help define the role of passive rGE by disentangling overlapping genetic, shared, and non-shared environmental effects [65].

The influence of shared environmental effects on childhood trauma’s covariance with high-risk for BD may have been masked by the presence of non-additive genetic effects, as these are confounded in twin models [66]. Nevertheless, the lack of effects may indicate etiological differences (related to shared environmental factors) in childhood trauma’s association with subclinical hypomania based on the latter’s severity.

Non-shared environmental factors were minor contributors to childhood trauma’s covariation with both measures of hypomania, but CIs overlapped with zero, and thus lacked statistical significance, in the context of high-risk for BD. Given the limited sample size of our “high-risk for BD” group, it would be important for studies with greater power to replicate these results. While not direct evidence for causality, our finding showing a small non-shared environmental overlap does not rule out causal effects of childhood trauma on hypomania as a quantitative trait [67].

### Gene–environment interplay between polygenic scores, childhood trauma, and subclinical hypomania

We provide further evidence of rGE between childhood trauma and PGS for several conditions: MDD, schizophrenia, ADHD, anxiety, and PTSD [15, 16, 68]. Genetic liability for these conditions may partly explain some of the active/evocative rGE suggested by the bivariate twin models, particularly MDD, as this PGS showed the most pronounced and consistent effect across models.

A significant correlation in the opposite direction was detected between the BD-II PGS and childhood trauma in the high-risk for BD group. This finding is consistent with those from a study that used a clinical BD sample [18]. The reason for this negative correlation is unclear, but one possible explanation is that individuals at high-risk for BD with a genetic predisposition for BD may evoke/select themselves into *less* environmental adversity. For instance, the “bright” dimension of hypomania has been defined as socially advantageous, and some of these symptoms (e.g., elation) are more prevalent among high-risk individuals [2]. Nevertheless, both positive and negative rGE are likely to be underpinned by a more complex multifactorial interplay [18]. Other genes (e.g., PGS for childhood maltreatment) [69], environmental (socioeconomic status) [70], and protective factors (e.g., resilience) [71] linked to childhood trauma warrant further investigation in this context.

The final novel finding is that genetic risk for MDD was linked to increased susceptibility to childhood trauma’s effect on both hypomanic symptoms and high-risk for BD, with evidence of departure from additivity in relation to the latter. This is in line with the implication of biological mechanisms in the development of more severe cases and can be indicative of a particularly vulnerable group that would benefit most from intervention [53]. Similar GxE effects have been reported for depression [72], although these were contested by a more recent meta-analysis [73]. It is important that our results are replicated in subclinical hypomania and BD samples to be conclusive.

### Implications

These initial findings increase our understanding of childhood trauma’s role in the etiology of subclinical hypomania, showing considerable genetic effects in the form of i. bivariate heritability, ii. genetic overlap, iii. rGE, and iv. GxE with psychiatric and neurodevelopmental PGS. This highlights the importance that future research testing the influence of childhood trauma account for the impact of complex gene–environment interplay using a range of genetically sensitive designs. While high polygenic risk for MDD was found to increase vulnerability to childhood trauma’s effect on both measures of hypomania, there was evidence of departure from additivity in the context of high-risk for BD. This finding is compatible with the implication of biological processes and underscores this group’s potential as a target for research (e.g., phenotypic definition and examination of developmental pathways to psychiatric conditions, including BD) and clinical efforts (e.g., prevention and intervention) [49].

More broadly, our findings linking childhood trauma and hypomania to genetic risk for various conditions are consistent with the evidence that psychiatric and adversity phenotypes share a genetic basis [25]. This reinforces the relevance of examining the influence of different PGS, and not only those measuring a specific trait of interest. Finally, it must be noted that evidence of genetic effects does not imply that those who experience childhood trauma are responsible for their own victimization (e.g., neither as active nor as evocative rGE), or that parental mental illness is inherently linked to offspring childhood trauma. Rather, it suggests that the association between trauma and hypomania may not be causal, underscoring the need to account for genetic characteristics to improve the course and outcomes of psychiatric phenotypes [74], such as hypomania. Individuals presenting with symptoms of hypomania may be at increased risk of experiencing childhood events as traumatic and may benefit from additional assessment and clinical support targeting the impact of childhood trauma.

### Methodological considerations

The main strength of this study is the use of a large genetically informed twin sample, permitting the examination of genetic and environmental influences on the association between childhood trauma and subclinical hypomania using twin and polygenic data. There are also limitations that should be considered when interpreting the findings. Firstly, childhood trauma was assessed retrospectively using a self-report measure. Retrospective self-reports of childhood trauma are robust predictors of psychiatric problems and have shown good reliability and validity among individuals with BD [75], but they can be vulnerable to bias (e.g., recall bias).

Secondly, the present study only focused on recollection of emotional and physical maltreatment, but other overlapping types of trauma (e.g., emotional neglect and sexual abuse) are linked to psychopathology such as BD [7, 8] and need to be studied in the context of subclinical hypomania. Thirdly, the power of the PGS included in this study differs in terms of the quality and size of the GWAS they were derived from. For instance, our findings showing a nonsignificant rGE between the autism PGS and childhood trauma are not consistent with previous findings [15] and may be due to the smaller sample size of the GWAS used for this PGS [40], In addition, most GxE effects are small, requiring very large sample sizes to detect reliable effects [53, 76]. Our results should be interpreted with caution and replicated in a larger sample. Lastly, PGS data in TEDS were only available for participants of European ancestry, limiting the generalizability of findings. Diverse GWAS are needed to address this issue and improve power.

## Conclusion

In this study, childhood trauma was associated with hypomanic symptoms and being at high-risk for BD at age 26 years. These associations were partially driven by shared genetic factors with childhood trauma, particularly high-risk for BD. There was evidence of rGE with childhood trauma, explained by the PGS for MDD, schizophrenia, ADHD, anxiety, PTSD, and BD II. GxE were detected between childhood and the MDD PGS on both hypomanic symptoms and high-risk for BD. These findings offer support for childhood trauma as a risk factor for subclinical hypomania, while highlighting the influence of gene–environment interplay on these phenotypes and their association. These results do not imply that survivors of childhood trauma are responsible for their experiences via genetic mechanisms, but suggest that the association between trauma and hypomania may not be causal and emphasize the importance of accounting for genetic characteristics to improve hypomania’s course and outcomes.

## Supporting information

10.1192/j.eurpsy.2025.10117.sm001 Gonzalez-Calvo et al. supplementary material Gonzalez-Calvo et al. supplementary material

## Data Availability

TEDS data used within this study are accessible on request via an online proposal form. Please see https://www.teds.ac.uk/researchers/teds-data-access-policy/ for further details. Please note that the TEDS website contains details of all data that are available through a fully searchable data dictionary (https://datadictionary.teds.ac.uk/home.htm).

## Supplementary material

The supplementary material for this article can be found at http://doi.org/10.1192/j.eurpsy.2025.10117.

## References

[1] American Psychiatric Association. Diagnostic and statistical manual of mental disorders [Internet].5th ed. American Psychiatric Association; 2013 [cited 2023 Sep 20]. Available from: 10.1176/appi.books.9780890425596

[2] Hosang GM, Cardno AG, Freeman D, Ronald A. Characterization and structure of hypomania in a British nonclinical adolescent sample. J Affect Disord. 2017;207:228–35. 27728870 10.1016/j.jad.2016.08.033 PMC5113133

[3] Hosang GM, Martin J, Karlsson R, Lundström S, Larsson H, Ronald A, et al. Association of Etiological Factors for hypomanic symptoms, bipolar disorder, and other severe mental illnesses. JAMA Psychiatry. 2022;79(2):143. 34910090 10.1001/jamapsychiatry.2021.3654 PMC8674803

[4] Päären A, Bohman H, Von Knorring AL, Von Knorring L, Olsson G, Jonsson U. Hypomania spectrum disorder in adolescence: A 15-year follow-up of non-mood morbidity in adulthood. BMC Psychiatry. 2014;14(1):9. 24428938 10.1186/1471-244X-14-9 PMC3898212

[5] Martin J, Taylor MJ, Lichtenstein P. Assessing the evidence for shared genetic risks across psychiatric disorders and traits. Psychol Med. 2018;48(11):1759–74. 29198204 10.1017/S0033291717003440 PMC6088770

[6] Gonzalez-Calvo I, Ronald A, Shakoor S, Taylor MJ, Eley TC, Hosang GM. Perinatal risk factors and subclinical hypomania: A prospective community study. J Affect Disord. 2024;362:885–92. 39029678 10.1016/j.jad.2024.07.118

[7] Palmier-Claus JE, Berry K, Bucci S, Mansell W, Varese F. Relationship between childhood adversity and bipolar affective disorder: Systematic review and meta-analysis. Br J Psychiatry. 2016;209(6):454–9. 27758835 10.1192/bjp.bp.115.179655

[8] Hosang GM, Fisher HL, Hodgson K, Maughan B, Farmer AE. Childhood maltreatment and adult medical morbidity in mood disorders: Comparison of unipolar depression with bipolar disorder. Br J Psychiatry. 2018;213(5):645–53. 30232950 10.1192/bjp.2018.178 PMC6429240

[9] Gajwani R, Dinkler L, Lundström S, Lichtenstein P, Gillberg C, Minnis H. Mania symptoms in a Swedish longitudinal population study: The roles of childhood trauma and neurodevelopmental disorders. J Affect Disord. 2021;280:450–6. 33242716 10.1016/j.jad.2020.10.076

[10] Caspi A, Houts RM, Ambler A, Danese A, Elliott ML, Hariri A, et al. Longitudinal assessment of mental health disorders and comorbidities across 4 decades among participants in the Dunedin birth cohort study. JAMA Netw Open. 2020;3(4):e203221. 32315069 10.1001/jamanetworkopen.2020.3221 PMC7175086

[11] Kroon JS, Wohlfarth TD, Dieleman J, Sutterland AL, Storosum JG, Denys D, et al. Incidence rates and risk factors of bipolar disorder in the general population: A population-based cohort study. Bipolar Disord. 2013;15(3):306–13. 23531096 10.1111/bdi.12058

[12] Jay Schulz-Heik R, Rhee SH, Silvern L, Lessem JM, Haberstick BC, Hopfer C, et al. Investigation of genetically mediated child effects on maltreatment. Behav Genet. 2009;39(3):265–76. 19283463 10.1007/s10519-009-9261-4 PMC2693353

[13] Pittner K, Bakermans-Kranenburg MJ, Alink LRA, Buisman RSM, Van Den Berg LJM, Block LHCGCC, et al. Estimating the heritability of experiencing child maltreatment in an extended family design. Child Maltreat 2020;25(3):289–99. 31773993 10.1177/1077559519888587 PMC7370654

[14] Warrier V, Kwong ASF, Luo M, Dalvie S, Croft J, Sallis HM, et al. Gene–environment correlations and causal effects of childhood maltreatment on physical and mental health: A genetically informed approach. Lancet Psychiatry. 2021;8(5):373–86. 33740410 10.1016/S2215-0366(20)30569-1 PMC8055541

[15] Peel AJ, Purves KL, Baldwin JR, Breen G, Coleman JRI, Pingault JB, et al. Genetic and early environmental predictors of adulthood self-reports of trauma. Br J Psychiatry. 2022;221(4):613–20. 35105391 10.1192/bjp.2021.207

[16] Ratanatharathorn A, Koenen KC, Chibnik LB, Weisskopf MG, Rich-Edwards JW, Roberts AL. Polygenic risk for autism, attention-deficit hyperactivity disorder, schizophrenia, major depressive disorder, and neuroticism is associated with the experience of childhood abuse. Mol Psychiatry. 2021;26(5):1696–705. 33483690 10.1038/s41380-020-00996-w PMC8164961

[17] Woolway GE, Smart SE, Lynham AJ, Lloyd JL, Owen MJ, Jones IR, et al. Schizophrenia polygenic risk and experiences of childhood adversity: A systematic review and meta-analysis. Schizophr Bull. 2022;48(5):967–80. 35674151 10.1093/schbul/sbac049 PMC9434424

[18] Aas M, Bellivier F, Bettella F, Henry C, Gard S, Kahn J, et al. Childhood maltreatment and polygenic risk in bipolar disorders. Bipolar Disord. 2020;22(2):174–81. 31628696 10.1111/bdi.12851

[19] Plomin R, DeFries JC, Loehlin JC. Genotype-environment interaction and correlation in the analysis of human behavior. Psychol Bull. 1977;84(2):309–22. 557211

[20] Kendler KS, Eaves LJ. Models for the joint effect of genotype and environment on liability to psychiatric illness. Am J Psychiatry. 1986;143(3):279–89. 3953861 10.1176/ajp.143.3.279

[21] De Castro-Catala M, Van Nierop M, Barrantes-Vidal N, Cristóbal-Narváez P, Sheinbaum T, Kwapil TR, et al. Childhood trauma, BDNF Val66Met and subclinical psychotic experiences. Attempt at replication in two independent samples. J Psychiatr Res. 2016;83:121–9. 27596955 10.1016/j.jpsychires.2016.08.014

[22] Park YM, Shekhtman T, Kelsoe JR. Interaction between adverse childhood experiences and polygenic risk in patients with bipolar disorder. Transl Psychiatry. 2020;10(1):326. 32963226 10.1038/s41398-020-01010-1 PMC7509781

[23] Wilcox HC, Fullerton JM, Glowinski AL, Benke K, Kamali M, Hulvershorn LA, et al. Traumatic stress interacts with bipolar disorder genetic risk to increase risk for suicide attempts. J Am Acad Child Adolesc Psychiatry. 2017;56(12):1073–80. 29173741 10.1016/j.jaac.2017.09.428 PMC5797709

[24] Hosang GM, Martini MI, Ronald A, Larsson H, Lundström S, Lichtenstein P, Taylor MJ. Subclinical hypomania, psychiatric and neurodevelopmental diagnoses: Phenotypic and aetiological overlap. Journal of Child Psychology and Psychiatry, jcpp.70045. 10.1111/jcpp.70045 PMC13102046 40913366

[25] Selzam S, Coleman JRI, Caspi A, Moffitt TE, Plomin R. A polygenic p factor for major psychiatric disorders. Transl Psychiatry. 2018;8(1):205. 30279410 10.1038/s41398-018-0217-4 PMC6168558

[26] Lockhart C, Bright J, Ahmadzadeh Y, Breen G, Bristow S, Boyd A, et al. Twins early development study (TEDS): A genetically sensitive investigation of mental health outcomes in the mid-twenties. JCPP Adv. 2023;3(2):e12154. 37753150 10.1002/jcv2.12154 PMC10519737

[27] Haworth CMA, Davis OSP, Plomin R. Twins early development study (TEDS): A genetically sensitive investigation of cognitive and Behavioral development from childhood to Young adulthood. Twin Res Hum Genet. 2013;16(1):117–25. 23110994 10.1017/thg.2012.91 PMC3817931

[28] Rimfeld K, Malanchini M, Spargo T, Spickernell G, Selzam S, McMillan A, et al. Twins early development study: A genetically sensitive investigation into Behavioral and cognitive development from infancy to emerging adulthood. Twin Res Hum Genet. 2019;22(6):508–13. 31544730 10.1017/thg.2019.56 PMC7056571

[29] Houtepen LC, Heron J, Suderman MJ, Tilling K, Howe LD. Adverse childhood experiences in the children of the Avon longitudinal study of parents and children (ALSPAC). Wellcome Open Res. 2018;3:106. 30569020 10.12688/wellcomeopenres.14716.1 PMC6281007

[30] Wagner KD, Hirschfeld RMA, Emslie GJ, Findling RL, Gracious BL, Reed ML. Validation of the mood disorder questionnaire for bipolar disorders in adolescents. J Clin Psychiatry. 2006;67(05):827–30. 16841633 10.4088/jcp.v67n0518

[31] Youngstrom EA, Genzlinger JE, Egerton GA, Van Meter AR. Multivariate meta-analysis of the discriminative validity of caregiver, youth, and teacher rating scales for pediatric bipolar disorder: Mother knows best about mania. Arch Sci Psychol. 2015;3(1):112–37.

[32] Hirschfeld RMA, Williams JBW, Spitzer RL, Calabrese JR, Flynn L, Keck PE, et al. Development and validation of a screening instrument for bipolar Spectrum disorder: The mood disorder questionnaire. Am J Psychiatry. 2000;157(11):1873–5. 11058490 10.1176/appi.ajp.157.11.1873

[33] Davies MR, Kalsi G, Armour C, Jones IR, McIntosh AM, Smith DJ, et al. The genetic links to anxiety and depression (GLAD) study: Online recruitment into the largest recontactable study of depression and anxiety. Behav Res Ther. 2019;123:103503. 31715324 10.1016/j.brat.2019.103503 PMC6891252

[34] Selzam S, McAdams TA, Coleman JRI, Carnell S, O’Reilly PF, Plomin R, et al. Evidence for gene-environment correlation in child feeding: Links between common genetic variation for BMI in children and parental feeding practices. PLoS Genet. 2018;14(11):e1007757. 30457987 10.1371/journal.pgen.1007757 PMC6245504

[35] TEDS DNA Studies [Internet]. [cited 2025 Feb 24]. Available from: https://datadictionary.teds.ac.uk/studies/dna.htm

[36] Mullins N, Forstner AJ, O’Connell KS, Coombes B, Coleman JRI, Qiao Z, et al. Genome-wide association study of more than 40,000 bipolar disorder cases provides new insights into the underlying biology. Nat Genet. 2021;53(6):817–29. 34002096 10.1038/s41588-021-00857-4 PMC8192451

[37] eQTLGen, 23andMe, the Major Depressive Disorder Working Group of the Psychiatric Genomics Consortium, Wray NR, Ripke S, Mattheisen M, et al. Genome-wide association analyses identify 44 risk variants and refine the genetic architecture of major depression. Nat Genet. 2018;50(5):668–81. 29700475 10.1038/s41588-018-0090-3 PMC5934326

[38] Trubetskoy V, Pardiñas AF, Qi T, Panagiotaropoulou G, Awasthi S, Bigdeli TB, et al. Mapping genomic loci implicates genes and synaptic biology in schizophrenia. Nature. 2022;604(7906):502–8. 35396580 10.1038/s41586-022-04434-5 PMC9392466

[39] Nievergelt CM, Maihofer AX, Klengel T, Atkinson EG, Chen CY, Choi KW, et al. International meta-analysis of PTSD genome-wide association studies identifies sex- and ancestry-specific genetic risk loci. Nat Commun. 2019;10(1):4558. 31594949 10.1038/s41467-019-12576-w PMC6783435

[40] The Autism Spectrum Disorders Working Group of The Psychiatric Genomics Consortium. Meta-analysis of GWAS of over 16,000 individuals with autism spectrum disorder highlights a novel locus at 10q24.32 and a significant overlap with schizophrenia. Mol Autism. 2017;8(1):21. 28540026 10.1186/s13229-017-0137-9 PMC5441062

[41] Purves KL, Coleman JRI, Meier SM, Rayner C, Davis KAS, Cheesman R, et al. A major role for common genetic variation in anxiety disorders. Mol Psychiatry. 2020;25(12):3292–303. 31748690 10.1038/s41380-019-0559-1 PMC7237282

[42] Demontis D, Walters GB, Athanasiadis G, Walters R, Therrien K, Nielsen TT, et al. Genome-wide analyses of ADHD identify 27 risk loci, refine the genetic architecture and implicate several cognitive domains. Nat Genet. 2023;55(2):198–208. 36702997 10.1038/s41588-022-01285-8 PMC10914347

[43] Jiang X, Zai CC, Dimick MK, Kennedy JL, Young LT, Birmaher B, et al. Psychiatric polygenic risk scores across youth with bipolar disorder, youth at high risk for bipolar disorder, and controls. J Am Acad Child Adolesc Psychiatry. 2024;63(11):1149–57. 38340895 10.1016/j.jaac.2023.12.009

[44] Richards AL, Cardno A, Harold G, Craddock NJ, Di Florio A, Jones L, et al. Genetic liabilities differentiating bipolar disorder, schizophrenia, and major depressive disorder, and phenotypic heterogeneity in bipolar disorder. JAMA Psychiatry. 2022;79(10):1032. 36044200 10.1001/jamapsychiatry.2022.2594 PMC9434480

[45] Forty L, Kelly M, Jones L, Jones I, Barnes E, Caesar S, et al. Reducing the hypomania checklist (HCL-32) to a 16-item version. J Affect Disord. 2010;124(3):351–6. 20129673 10.1016/j.jad.2010.01.004

[46] Calvo IG, Ronald A, Taylor M, Havers L, Hosang G. Association between psychosocial risk factors and youth subclinical hypomania [Internet]. OSF Registries; 2024 [cited 2025 Mar 26]. Available from: https://osf.io/pvqda/

[47] Zetterqvist J, Sjölander A. Doubly robust estimation with the R package drgee. Epidemiol Methods [Internet]. 2015 Jan 1 [cited 2024 Mar 1];4(1). Available from: https://www.degruyter.com/document/doi/10.1515/em-2014-0021/html

[48] Hadianfard H. Child abuse in group of children with attention deficit-hyperactivity disorder in comparison with normal children. Int J Community Based Nurs Midwifery. 2014;2(2):77–84. 25349848 PMC4201192

[49] Ronald A, Sieradzka D, Cardno AG, Haworth CMA, McGuire P, Freeman D. Characterization of psychotic experiences in adolescence using the specific psychotic experiences questionnaire: Findings from a study of 5000 16-year-old twins. Schizophr Bull. 2014;40(4):868–77. 24062593 10.1093/schbul/sbt106 PMC4059437

[50] Knopik VS, Neiderheiser J, DeFries JC, Plomin R. Behavioral genetics. 7th ed. New York: Worth; 2017.

[51] Hosang GM, Shakoor S, King N, Sanches M, Vincent JB, Kennedy JL, et al. Interplay between polygenic risk for mood disorders and stressful life events in bipolar disorder. J Affect Disord. 2024;350:565–72. 38246285 10.1016/j.jad.2024.01.167

[52] Mullins N, Power RA, Fisher HL, Hanscombe KB, Euesden J, Iniesta R, et al. Polygenic interactions with environmental adversity in the aetiology of major depressive disorder. Psychol Med. 2016;46(4):759–70. 26526099 10.1017/S0033291715002172 PMC4754832

[53] VanderWeele TJ, Knol MJ. A tutorial on interaction. Epidemiol Methods [Internet]. 2014 Jan 1 [cited 2024 Nov 26];3(1). Available from: https://www.degruyter.com/document/doi/10.1515/em-2013-0005/html

[54] Keller MC. Gene × environment interaction studies have not properly controlled for potential confounders: The problem and the (simple) solution. Biol Psychiatry. 2014;75(1):18–24. 24135711 10.1016/j.biopsych.2013.09.006 PMC3859520

[55] Etain B, Henry C, Bellivier F, Mathieu F, Leboyer M. Beyond genetics: Childhood affective trauma in bipolar disorder. Bipolar Disord. 2008;10(8):867–76. 19594502 10.1111/j.1399-5618.2008.00635.x

[56] Ackner S, Skeate A, Patterson P, Neal A. Emotional abuse and psychosis: A recent review of the literature. J Aggress Maltreatment Trauma. 2013;22(9):1032–49.

[57] Aas M, Henry C, Andreassen OA, Bellivier F, Melle I, Etain B. The role of childhood trauma in bipolar disorders. Int J Bipolar Disord. 2016;4(1):2. 26763504 10.1186/s40345-015-0042-0 PMC4712184

[58] Heim CM, Mayberg HS, Mletzko T, Nemeroff CB, Pruessner JC. Decreased cortical representation of genital somatosensory field after childhood sexual abuse. Am J Psychiatry. 2013;170(6):616–23. 23732967 10.1176/appi.ajp.2013.12070950

[59] Kurtz M, Mohring P, Förster K, Bauer M, Kanske P. Deficits in explicit emotion regulation in bipolar disorder: A systematic review. Int J Bipolar Disord. 2021;9(1):15. 33937951 10.1186/s40345-021-00221-9 PMC8089068

[60] Sartor CE. Common heritable contributions to low-risk trauma, high-risk trauma, posttraumatic stress disorder, and major depression. Arch Gen Psychiatry. 2012;69(3):293. 22393221 10.1001/archgenpsychiatry.2011.1385 PMC3594801

[61] South SC, Schafer MH, Ferraro KF. Genetic and environmental overlap between childhood maltreatment and adult physical health. Twin Res Hum Genet. 2015;18(5):533–44. 26379062 10.1017/thg.2015.62 PMC6058311

[62] Barkley RA. Major life activity and health outcomes associated with attention-deficit/hyperactivity disorder. J Clin Psychiatry. 2002;63(Suppl 12):10–5. 12562056

[63] Bastos RA, Campos LS, Faria-Schützer DB, Brito ME, Da Silva DR, Dos Santos-Junior A, et al. Offspring of mothers with bipolar disorder: A systematic review considering personality features. Braz J Psychiatry. 2022;44(1):94–102. 35170672 10.1590/1516-4446-2020-1465 PMC8827366

[64] Reupert A, Maybery D. Families affected by parental mental illness: A multiperspective account of issues and interventions. Am J Orthopsychiatry. 2007;77(3):362–9. 17696664 10.1037/0002-9432.77.3.362

[65] Plomin R, Loehlin JC, DeFries JC. Genetic and environmental components of ‘environmental’ influences. Dev Psychol. 1985;21(3):391–402.

[66] Rettew DC, Rebollo-Mesa I, Hudziak JJ, Willemsen G, Boomsma DI. Non-additive and additive genetic effects on extraversion in 3314 Dutch adolescent twins and their parents. Behav Genet. 2008;38(3):223–33. 18240014 10.1007/s10519-008-9192-5 PMC3319035

[67] Bornovalova MA, Huibregtse BM, Hicks BM, Keyes M, McGue M, Iacono W. Tests of a direct effect of childhood abuse on adult borderline personality disorder traits: A longitudinal discordant twin design. J Abnorm Psychol. 2013;122(1):180–94. 22686871 10.1037/a0028328 PMC3482426

[68] ter Kuile AR, Hübel C, Cheesman R, Coleman JRI, Peel AJ, Levey DF, et al. Genetic decomposition of the heritable component of reported childhood maltreatment. Biol Psychiatry Glob Open Sci. 2023;3(4):716–24. 37881567 10.1016/j.bpsgos.2023.03.003 PMC10593925

[69] Dalvie S, Maihofer AX, Coleman JRI, Bradley B, Breen G, Brick LA, et al. Genomic influences on self-reported childhood maltreatment. Transl Psychiatry. 2020;10(1):38. 32066696 10.1038/s41398-020-0706-0 PMC7026037

[70] Walsh D, McCartney G, Smith M, Armour G. Relationship between childhood socioeconomic position and adverse childhood experiences (ACEs): A systematic review. J Epidemiol Community Health. 2019;73(12):1087–93. 31563897 10.1136/jech-2019-212738 PMC6872440

[71] Park JY, Lee CW, Jang Y, Lee W, Yu H, Yoon J, et al. Relationship between childhood trauma and resilience in patients with mood disorders. J Affect Disord. 2023;323:162–70. 36395993 10.1016/j.jad.2022.11.003

[72] Peyrot WJ, Milaneschi Y, Abdellaoui A, Sullivan PF, Hottenga JJ, Boomsma DI, et al. Effect of polygenic risk scores on depression in childhood trauma. Br J Psychiatry. 2014;205(2):113–9. 24925986 10.1192/bjp.bp.113.143081 PMC4118052

[73] Peyrot WJ, Van Der Auwera S, Milaneschi Y, Dolan CV, Madden PAF, Sullivan PF, et al. Does childhood trauma moderate polygenic risk for depression? A meta-analysis of 5765 subjects from the psychiatric genomics consortium. Biol Psychiatry. 2018;84(2):138–47. 29129318 10.1016/j.biopsych.2017.09.009 PMC5862738

[74] Andreassen OA, Hindley GFL, Frei O, Smeland OB. New insights from the last decade of research in psychiatric genetics: Discoveries, challenges and clinical implications. World Psychiatry Off J World Psychiatr Assoc WPA. 2023;22(1):4–24. 10.1002/wps.21034 PMC9840515 36640404

[75] Hosang GM, Manoli A, Shakoor S, Fisher HL, Parker C. Reliability and convergent validity of retrospective reports of childhood maltreatment by individuals with bipolar disorder. Psychiatry Res. 2023;321:115105. 36796256 10.1016/j.psychres.2023.115105

[76] Dudbridge F. Power and predictive accuracy of polygenic risk scores. PLoS Genet. 2013;9(3):e1003348. 23555274 10.1371/journal.pgen.1003348 PMC3605113

