# Supplementary material for: Childhood trauma and subclinical hypomania in early adulthood: A genetically informative study
Source: Eur Psychiatry. 2025 Oct 10;68(1):e157. doi: 10.1192/j.eurpsy.2025.10117 (PMC12646032; doi:10.1192/j.eurpsy.2025.10117)
Supplement: Gonzalez-Calvo et al. supplementary material [file S092493382510117Xsup001.docx]

**Supplementary materials**

Table of Contents

[Supplementary measures. Hypomania Checklist-16 (HCL-16, Forty et al., 2010) 2](#_Toc206773992)

[Supplementary Table 1. Intraclass twin correlations and number of participants 3](#_Toc206773993)

[Supplementary Table 2. Fit Statistics and Parameter Estimates for Univariate Models: Retrospective childhood trauma (total score), subclinical hypomania, and high-risk for bipolar disorder 4](#_Toc206773994)

[Supplementary Table 3. Fit Statistics and Parameter Estimates for Best Fitting Bivariate Models 5](#_Toc206773995)

[Supplementary Table 4. Associations between polygenic scores, hypomanic symptoms and high-risk for bipolar disorder 6](#_Toc206773996)

[Supplementary Table 5. Correlation estimates between childhood trauma, polygenic scores and principal components of ancestry 7](#_Toc206773997)

[Supplementary Table 6. Correlation estimates between childhood trauma, polygenic scores and principal components of ancestry 8](#_Toc206773998)

# ***Supplementary measures.*** Hypomania Checklist-16 (HCL-16, Forty et al., 2010)

The HCL-16 is based on the longer HCL-32 version (Angst et al., 2005). This instrument has good reliability (Forty et al., 2010). The HCL-16 asks participants to recall any “high” period in their lives (i.e., when they felt unusually elevated levels of energy, activity, and mood), and indicate the presence of 16 hypomania-related behaviours, thoughts, and emotions (e.g., ‘I take more risks in my daily life (in my work and/or other activities’) using yes/no responses. In the current study, one item was removed: ‘I feel more flirtatious and/or am more sexually active’ to avoid offending participants (Hosang et al., 2017). Total HCL-16 scores range from 0 to 15. This instrument also includes items that ask about the duration and impairment caused by the “high” periods overall. Participants are classified as being at high-risk for BD if they (1) had a HCL-16 score of 8 or more (cut-off), (2) that lasted at least 2 days, and (3) were associated with any type of negative impact or reaction, in line with previously used categorisations (Hosang et al., 2017).

# ***Supplementary Table 1***. Intraclass twin correlations and number of participants

|  | **MZ**  **ICC (95% CI)** | **DZ**  **ICC (95% CI)** | **Number of individuals** | | |
| --- | --- | --- | --- | --- | --- |
| **Univariate twin correlations** |  |  | **MZ** | **DZ** |  |
| Childhood trauma | 0.65 (0.61 – 0.68) | 0.40 (0.36 – 0.43) | 2696 | 3996 |  |
| Hypomanic symptoms at age 26 | 0.35 (0.29 – 0.40) | 0.21 (0.16 – 0.25) | 2180 | 3084 |  |
| High-risk for bipolar disorder at age 26 | 0.43 (0.20 – 0.63) | 0.14 (-0.12 – 0.37) | 2162 | 3042 |  |
| **Cross-trait cross-twin correlation** |  |  |  |  |  |
| Childhood trauma and hypomanic symptoms | 0.19 (0.13 – 0.24) | 0.13 (0.08 – 0.18) | 2212 | 3284 |  |
| Childhood trauma and high-risk for bipolar disorder | 0.16 (0.10 – 0.22) | 0.03 (-0.02 – 0.09) | 2162 | 3042 |  |

Intraclass correlations using transformed standardized age and sex regressed scales.

Abbreviations: ICC (intraclass correlations), CI (Confidence Intervals)

# ***Supplementary Table 2***. Fit Statistics and Parameter Estimates for Univariate Models: Retrospective childhood trauma (total score), subclinical hypomania, and high-risk for bipolar disorder

| Univariate Model |  |  | |  |  | |  |  |  | |  | |  | | | |  |  |
| --- | --- | --- | --- | --- | --- | --- | --- | --- | --- | --- | --- | --- | --- | --- | --- | --- | --- | --- |
|  | Model fit | | |  |  | |  |  |  | |  | | |  | | |  |  |
|  |  | ACE^1^ model compared to saturated model  AE and CE models compared to ACE model | | | | | | | | | | | | | Parameter estimates | | |  |
|  | Model | ep | -2ll | d.f. | LRT | Δ*df* | | AIC | BIC | *P* | | A^2^ | | | | C^2^ | | E^2^ |
| Childhood trauma |  |  |  |  |  |  | |  |  |  | |  | | | |  | |  |
|  | Sat | 10 | 19261.91 | 8371 |  |  | | - | - |  | |  | | | |  | |  |
|  |  |  |  |  |  |  | |  |  |  | |  | | | |  | |  |
|  | **ACE** | **4** | **19273.47** | **8377** | **11.56** | **6** | | **19281.47** | **-60088.39** | **.07** | | **0.46**  **(0.39-0.51)** | | | | **0.18**  **(0.13-0.23)** | | **0.36**  **(0.34-0.38)** |
|  | AE | 3 | 19293.42 | 8378 | 19.95 | 1 | | 19299.42 | -60077.91 | <.0001 | | 0.65  (0.62-0.68) | | | |  | | 0.35  (0.33-0.37) |
|  | CE | 3 | 19376.82 | 8378 | 103.35 | 1 | | 19382.82 | -59994.51 | <.0001 | |  | | | | 0.51  (0.49-0.54) | | 0.49  (0.47-0.51) |
| Hypomanic symptoms at age 26 |  |  |  |  |  |  | |  |  |  | |  | | | |  | |  |
|  | Sat | 10 | 25290.57 | 7625 |  |  | | - | - |  | |  | | | |  | |  |
|  | ACE | 4 | 25302.54 | 7631 | 11.97 | 6 | | 25310.53 | -46991.88 | .07 | | 0.29  (0.25-0.50) | | | | 0.06  (-0.02-0.18) | | 0.65  (0.58-0.68) |
|  | **AE** | **3** | **25303.92** | **7632** | **1.38** | **1** | | **25309.92** | **-46999.97** | **.24** | | **0.37**  **(0.33-0.40)** | | | |  | | **0.63**  **(0.60-0.67)** |
|  | CE | 3 | 25319.09 | 7632 | 16.56 | 1 | | 25325.09 | -46984.80 | <.0001 | |  | | | | 0.27  (0.23-0.29) | | 0.73  (0.71-0.77) |
| High-risk for bipolar disorder at age 26 |  |  |  |  |  |  | |  |  |  | | A^2^ | | | | D^2^ | | E^2^ |
|  | Sat | 6 | 2797.34 | 7581 |  |  | |  | - |  | |  | | | |  | |  |
|  | ADE | 4 | 2799.06 | 7584 | 1.73 | 1 | | 2807.06 | -69050.09 | .63 | | 0.07  (-0.95 – 1.02) | | | | 0.38  (-0.66 – 1.46) | | 0.55  (0.36 – 0.79) |
|  | **AE** | **3** | **2799.55** | **7585** | 0.49 | **1** | | **2805.55** | **-69059.08** | **.49** | | **0.42**  **(0.20 – 0.60)** | | | |  | | **0.58**  **(0.40 – 0.80)** |

Please note: The ACE model is a statistical model frequently used to analyse twin data. This term is commonly used in twin studies and differs from ACEs (Adverse Childhood Experiences)

|  |
| --- |

# ***Supplementary Table 3.*** Fit Statistics and Parameter Estimates for Best Fitting Bivariate Models

| **Bivariate Model** |  |  | |  |  |  |  |  |  |
| --- | --- | --- | --- | --- | --- | --- | --- | --- | --- |
|  | **Model fit** |  | |  |  |  |  |  |  |
|  | **Model** | **ep** | **-2ll** | **d.f.** | **LRT** | **Δ*df*** | **AIC** | **BIC** | **P** |
| **Childhood trauma**  **and hypomanic symptoms** |  | ACE model compared to saturated model  AE and CE models compared to ACE model | | | |  |  |  |  |
|  | Sat | 28 | 44234.93 | 15988 |  |  | - | - |  |
|  | **ACE** | **11** | **44265.92** | **16005** | **30.98** | **17** | **44287.92** | **-107361.95** | **.23** |
|  | AE | 8 | 44287.91 | 16008 | 21.99 | 3 | 44303.91 | -107368.42 | <.001 |
|  | CE | 8 | 44387.86 | 16008 | 121.94 | 3 | 44403.86 | -107268.4 | <.001 |
| **Childhood trauma**  **and high-risk for bipolar disorder** |  | ADE model compared to saturated model  AE model compared to ADE model | | | | |  |  |  |
|  | Sat | 24 | 21936.19 | 15944 |  |  | - | - |  |
|  | ADE | 12 | 22443.63 | 15957 | 507.44 | 13 | 22467.63 | -128729.49 | <.0001 |
|  | **AE** | **9** | **22529.56** | **15960** | **85.92** | **3** | **22547.56** | **-107368.42** | **<.0001** |
|  | Parameter estimates for Best Fitting Bivariate Models | | | | | | | |  |
| **Childhood trauma**  **and hypomanic symptoms** | **ACE** | **Bivariate *a*2** | | **Bivariate c2** | **Bivariate e2** | ***r*a** | ***r_c_*** | ***r*e** |  |
|  |  | 0.51  (0.26–0.77) | | 0.32  (0.11–0.52) | 0.17  (0.08–0.25) | 0.31  (0.16 – 0.33) | 0.74  (0.71 – 0.76) | 0.08  (0.04 – 0.12) |  |
| **Childhood trauma**  **and high-risk for bipolar disorder** | **AE** | **Bivariate *a*2** | | **Bivariate e2** |  | ***r*a** | ***r*e** |  |  |
|  |  | 0.90  (0.73–1.08) | | 0.10  (-0.08–0.26) |  | 0.58  (0.41 – 0.80) | 0.09  (-0.07 – 0.26) |  |  |

Bivariate genetic (Bivariate *a*2), common environment (Bivariate *c*2), and unique environment (Bivariate *e*2) estimates indicate the proportion of phenotypic correlations explained by genetics, common, and unique environment, respectively. Bivariate genetic (*r*a), common environment (*r*c), and unique environment (*r*e) correlations indicate the genetic and environmental overlap between retrospective childhood trauma and hypomanic symptoms at age 26. 95% confidence intervals in parentheses

# ***Supplementary Table 4.*** Associations between polygenic scores, hypomanic symptoms and high-risk for bipolar disorder

|  | **Hypomanic symptoms at age 26** | | | | | | | | |  | | **High-risk for bipolar disorder at age 26** | | | | | | | | |  |
| --- | --- | --- | --- | --- | --- | --- | --- | --- | --- | --- | --- | --- | --- | --- | --- | --- | --- | --- | --- | --- | --- |
|  | *Univariable models* | | | | | *Multi-PGS model* | | | |  | | *Univariable models* | | | | | *Multi-PGS model* | | | | |
|  | **β** | **95% CI** | ***P*** |  | **β** | | **95% CI** | ***P*** |  | | **OR** | | **95% CI** | ***P*** |  | **OR** | | **95% CI** | ***P*** |  |  |
| Bipolar disorder PGS | 0.03 | 0.0008 – 0.06 | .05 |  | 0.01 | | -0.04 – 0.06 | .75 |  | | 1.11 | | 0.96 – 1.29 | .24 |  | 1.01 | | 0.86 – 1.19 | .88 |  |  |
| Bipolar disorder I PGS | 0.02 | -0.041– 0.05 | .26 |  | -0.02 | | -0.07 – 0.02 | .64 |  | | 1.06 | | 0.91 – 1.22 | .46 |  | 0.98 | | 0.83 – 1.15 | .80 |  |  |
| Bipolar disorder  **II PGS** | **0.04** | **0.02 – 0.07** | **.004** |  | 0.03 | | -0.01 – 0.07 | .36 |  | | 1.08 | | 0.93 – 1.25 | .34 |  | 1.01 | | 0.87 – 1.18 | .90 |  |  |
| **MDD PGS** | **0.06** | **0.03 – 0.09** | **.0001** |  | 0.02 | | -0.03 – 0.06 | .64 |  | | **1.38** | | **1.19 – 1.61** | **.0001** |  | **1.29** | | **1.11 – 1.51** | **.001** |  |  |
| **Schizophrenia PGS** | **0.05** | **0.01 – 0.07** | **.004** |  | -0.01 | | -0.05 – 0.03 | .75 |  | | 1.08 | | 0.93 – 1.25 | .34 |  | 1.00 | | 0.85 – 1.18 | .97 |  |  |
| **ADHD PGS** | **0.12** | **0.09 – 0.18** | **<.00001** |  | **0.13** | | **0.08 – 0.17** | **<.00001** |  | | **1.22** | | **1.046– 1.41** | **.02** |  | 1.11 | | 0.926– 1.29 | .15 |  |  |
| Autism PGS | 0.008 | -0.02 – 0.04 | .58 |  | -0.007 | | -0.05 – 0.03 | .75 |  | | 0.89 | | 0.78 – 1.02 | .18 |  | 0.88 | | 0.77 – 1.00 | .05 |  |  |
| **Anxiety PGS** | **0.06** | **0.03 – 0.09** | **.0002** |  | 0.03 | | -0.01 – 0.07 | .36 |  | | **1.22** | | **1.06 – 1.42** | **.01** |  | 1.12 | | 0.96 – 1.30 | .14 |  |  |
| **PTSD PGS** | **0.07** | **0.04 – 0.10** | **<.00001** |  | **0.05** | | **0.01 – 0.09** | **.04** |  | | **1.29** | | **1.11 – 1.50** | **.004** |  | **1.21** | | **1.00– 1.41** | **.01** |  |  |

*P*-values are reported FDR-adjusted (Benjamini-Hochberg procedure); β = Standardised beta; OR = Odds ratio. Two multi-PGS models were fitted (one including the overall BD PGS and the other the BD I PGS and BD II PGS) to avoid multicollinearity.

# ***Supplementary Table 5.*** Correlation estimates between childhood trauma, polygenic scores and principal components of ancestry

|  | 1 | 2 | 3 | 4 | 5 | 6 | 7 | 8 | 9 | 10 | 11 | 12 | 13 | 14 | 15 | 16 | 17 | 18 | 19 |
| --- | --- | --- | --- | --- | --- | --- | --- | --- | --- | --- | --- | --- | --- | --- | --- | --- | --- | --- | --- |
| 1. Childhood trauma |  |  |  |  |  |  |  |  |  |  |  |  |  |  |  |  |  |  |  |
| 2. Bipolar disorder PGS | 0.03 |  |  |  |  |  |  |  |  |  |  |  |  |  |  |  |  |  |  |
| 3. Bipolar disorder I PGS | 0.02 |  |  |  |  |  |  |  |  |  |  |  |  |  |  |  |  |  |  |
| 4. Bipolar disorder II PGS | 0.02 |  |  |  |  |  |  |  |  |  |  |  |  |  |  |  |  |  |  |
| 5. MDD PGS | 0.07 |  |  |  |  |  |  |  |  |  |  |  |  |  |  |  |  |  |  |
| 6. Schizophrenia PGS | 0.06 |  |  |  |  |  |  |  |  |  |  |  |  |  |  |  |  |  |  |
| 7. ADHD PGS | 0.1 |  |  |  |  |  |  |  |  |  |  |  |  |  |  |  |  |  |  |
| 8. Autism PGS | 0.03 |  |  |  |  |  |  |  |  |  |  |  |  |  |  |  |  |  |  |
| 9. Anxiety PGS | 0.05 |  |  |  |  |  |  |  |  |  |  |  |  |  |  |  |  |  |  |
| 10. PTSD PGS | 0.07 |  |  |  |  |  |  |  |  |  |  |  |  |  |  |  |  |  |  |
| 11. PC1 | -0.01 | -0.02 | -0.02 | -0.03 | 0.02 | -0.03 | 0.02 | -0.02 | -0.01 | 0.04 |  |  |  |  |  |  |  |  |  |
| 12. PC2 | 0.01 | 0.08 | 0.08 | 0.05 | 0.07 | 0.07 | 0.05 | -0.03 | 0.01 | 0.04 | 0 |  |  |  |  |  |  |  |  |
| 13. PC3 | 0.01 | 0.03 | 0 | 0.02 | 0 | 0.01 | 0.01 | 0.01 | 0 | 0.02 | -0.02 | 0 |  |  |  |  |  |  |  |
| 14. PC4 | -0.01 | -0.02 | -0.01 | -0.01 | -0.01 | 0 | 0 | -0.02 | -0.02 | 0 | 0.01 | -0.01 | 0.01 |  |  |  |  |  |  |
| 15. PC5 | -0.02 | 0.02 | 0.01 | 0.05 | 0.02 | 0 | 0 | 0.01 | 0 | 0.03 | -0.01 | 0.02 | 0 | -0.02 |  |  |  |  |  |
| 16. PC6 | 0 | 0 | 0 | -0.02 | 0.04 | 0.02 | 0 | 0 | 0.01 | 0.02 | -0.02 | 0.03 | 0.03 | -0.01 | 0 |  |  |  |  |
| 17. PC7 | 0.01 | -0.03 | -0.02 | -0.03 | 0 | -0.03 | -0.03 | 0.01 | -0.03 | -0.03 | -0.01 | 0 | 0.02 | -0.03 | -0.01 | 0.01 |  |  |  |
| 18. PC8 | -0.02 | -0.02 | -0.03 | 0 | -0.02 | -0.03 | -0.02 | -0.04 | -0.02 | 0 | -0.02 | 0.02 | -0.01 | -0.03 | 0.01 | -0.03 | 0 |  |  |
| 19. PC9 | -0.02 | 0 | 0 | -0.02 | 0.03 | -0.02 | -0.01 | 0 | 0.01 | 0.02 | 0.02 | -0.01 | 0.01 | 0.04 | 0.01 | 0.01 | 0.02 | 0.03 |  |
| 20. PC10 | 0.01 | 0.02 | 0.01 | -0.01 | 0.04 | 0.01 | 0.02 | -0.03 | 0.01 | 0.03 | 0.01 | 0.02 | -0.02 | -0.03 | 0.01 | 0.02 | 0 | 0.01 | 0.01 |

# ***Supplementary Table 6.*** Correlation estimates between childhood trauma, polygenic scores and principal components of ancestry

| **Predictor variables** | **VIF** |
| --- | --- |
| PC1 | 1.00 |
| PC2 | 1.00 |
| PC3 | 1.00 |
| PC4 | 1.01 |
| PC5 | 1.00 |
| PC6 | 1.00 |
| PC7 | 1.00 |
| PC8 | 1.00 |
| PC9 | 1.00 |
| PC10 | 1.00 |
| Childhood trauma | 1.02 |
| Bipolar disorder PGS | 5.36 |
| Bipolar disorder I PGS | 4.35 |
| Bipolar disorder II PGS | 1.47 |
| MDD PGS | 1.15 |
| Schizophrenia PGS | 1.20 |
| ADHD PGS | 1.08 |
| Autism PGS | 1.01 |
| Anxiety PGS | 1.07 |
| PTSD PGS | 1.05 |
